# Supplementary material for: Behavior–biological mismatch in metabolic health: Evidence from South Korean adults before, during, and after COVID-19 (KNHANES 2019–2022)
Source: PLoS One. 2026 Mar 20;21(3):e0344918. doi: 10.1371/journal.pone.0344918 (PMC13004492; doi:10.1371/journal.pone.0344918)
Supplement: Attachment — Submitted filename: Response to Reviewers.docx [file pone.0344918.s002.docx]

**Response to Reviewer Comments**

We would like to thank the reviewer for the constructive and insightful comments that have helped us improve the quality of our manuscript. Below are our point-by-point responses:

**Review #1**

Reviewer #1: This manuscript addresses an important and timely topic with potential relevance to both clinical practice and public health. The study is generally well structured and presents its findings in a clear and logical manner. The research question is relevant, and the authors have provided sufficient background to justify the study. However, few issues should be addressed to improve the rigor and clarity of the manuscript.

The authors conclude that PA alone is insufficient for cardiometabolic protection. This conclusion is supported, but causal language should be avoided due to the cross-sectional design.I would recommend adding: “associations” instead of “indicate” or “demonstrate.”

Thank you for the suggestion. We agree that given the cross-sectional nature of the study, it is more appropriate to use language that emphasizes associations rather than direct causality. In accordance with this suggestion, we have replaced causal terms such as "indicate" and "demonstrate" with "show an association suggesting" or "suggest associations indicating" throughout the manuscript, particularly in the Abstract, Discussion, and Conclusion sections.

Abstract :

The persistence and rise of PA–NH after the pandemic show an association suggesting that adherence to PA guidelines alone is insufficient for cardiometabolic protection.

Discussion:

These findings suggest associations indicating that adherence to PA recommendations alone may not ensure cardiometabolic resilience.

Conclusion:

These findings suggest associations indicating that adherence to PA guidelines alone may not ensure cardiometabolic protection, particularly among older adults and individuals exposed to complex lifestyle or stress-related factors.

Also, there are minor grammatical errors—e.g., inconsistent tense in PA classification description.

We appreciate the reviewer's careful reading. We have thoroughly reviewed the "Physical activity (PA)" section in the Methods and ensured that the tense is consistent. Specifically, we have corrected the instances where the present tense was used and unified them into the past tense to describe the study procedures.

Aerobic PA was defined as having engaged in at least 150 minutes of moderate-intensity activity per week, at least 75 minutes of vigorous-intensity activity, or an equivalent combination of both. Walking was defined as having walked for at least 10 minutes per session, totaling 30 minutes or more per day for at least five days during the past week. Muscle-strengthening activity was defined as having performed exercises such as push-ups, sit-ups, dumbbell or barbell training, or weightlifting for at least two days during the past week.

We have conducted a final proofreading of the manuscript and tables. We have corrected the typo in Table 4 (e.g., changed "ge" to "Age") and the capitalization in Table 1 and Table 4. The numerical data in Table 2 have been consistently adjusted for decimal places(e.g., changed "23.78" to "23.8%", “76.22” to “76.2%”).
